# COOH-terminal truncations and site-directed mutations enhance thermostability and chaperone-like activity of porcine αB-crystallin

**Published:** 2009-07-28

**Authors:** Jiahn-Haur Liao, Jiahn-Shing Lee, Shih-Hsiung Wu, Shyh-Horng Chiou

**Affiliations:** 1 Institute of Biological Chemistry, Academia Sinica, Taipei, Taiwan; 2 Institute of Biochemical Sciences, National Taiwan University, Taipei, Taiwan; 3 Graduate Institute of Medicine, Kaohsiung Medical University, Kaohsiung, Taiwan; 4 Department of Ophthalmology, Chang-Gung Memorial Hospital, Taipei, Taiwan

## Abstract

**Purpose:**

The COOH-terminal extension segment of αB-crystallin, a member of small heat shock protein (sHSP) family, appears to be a flexible polypeptide segment susceptible to proteolytic truncation and modifications under physiological conditions. To investigate its role on the structure and chaperone-like activity, we constructed various mutants of porcine αB-crystallin with either COOH-terminal serial truncations or site-specific mutagenesis on the last two residues.

**Methods:**

The structures of these mutants were analyzed by circular dichroism (CD) spectroscopy, fluorescence spectra, mass spectrometry, Gel-permeation FPLC, and dynamic light-scattering spectrophotometry. Chaperone activity assays were performed under thermal and non-thermal stresses. The stability of proteins was examined by turbidity assays and CD spectra.

**Results:**

All mutants showed similar secondary and tertiary structural features to the wild-type αB-crystallin as revealed by circular dichroism. However, truncations of the COOH-terminal segment generated crystallin aggregates with a molecular size slightly smaller than that of the wild-type αB-crystallin. The deletion of 12 residues from the COOH-terminal end greatly reduced the solubility, thermostability, and chaperone activity of αB-crystallin. On the contrary, the truncation of only 10 residues or less resulted in increased thermostability and enhanced anti-aggregation chaperone activity of αB-crystallin, with a maximal effect occurring on elimination of the last two residues. Moreover, displacing the last two lysines with glutamates or other neutral amino acids tended to show even higher chaperone activity than the deletion mutants.

**Conclusions:**

Our study clearly demonstrated that both the length and electrostatic charge of the COOH-terminal segment play crucial roles in governing the structural stability and chaperone activity of αB-crystallin.

## Introduction

The traditional view that proteins fold spontaneously in vivo was revised upon the finding that many proteins in a living cell will not fold correctly without the assistance of molecular chaperones. In the cytosol of prokaryotic and eukaryotic cells, molecular chaperones of different structural classes form a network of pathways, leading to correct folding states for most proteins [1]. The heat shock protein (HSP) family is one of the first and most prevalent protein chaperones induced under heat and mechanochemical stresses in most animal and plant cells.

α-Crystallin extracted from the lens, exists in solution as polydisperse aggregates ranging from 600 to 1,200 kDa with an average molecular mass of approximately 800 kDa. There are two α-crystallins known as αA and αB. αA-Crystallin is usually the most abundant subunit in the lens, although the αA/αB ratio varies considerably among species. Both subunits are about 20 kDa in size and have 55-60% sequence similarity. They were previously regarded as lens-specific polypeptides and exclusively being structural protein in nature [2]. Ingolia and Craig [3] first reported that heat shock proteins of *Drosophila* showed similarity to mammalian α-crystallin. It was shown later that αB-crystallin is not restricted to the lens even though αA-Crystallin is highly specialized for expression in the lens [4]. In 1992 it was demonstrated that α-crystallin functioned as a molecular chaperone by suppressing the aggregation of other polypeptides in vitro [5]. While both αA and αB-crystallins are molecular chaperones, only αB-crystallin is a functional small heat-shock protein (sHSP) [6-8]. Both subunits possess largely β-sheet structures and consist of two domains and a flexible COOH-terminal extension [9,10], as revealed by NMR studies. However, in the absence of an α-crystallin subunit 3D structure, the detailed protective chaperone mechanism of α-crystallin remains elusive and a matter of controversy. Due to its large size and the lack of crystallographic data available, the detailed molecular packing in the quaternary structure of α-crystallin has yet to be established. Recently, the structure of a homologous sHSP (sHSP16.5) from *Methanococcus jannaschii*, a hyperthermophilic archaeon, has been solved to 0.29 nm resolution [11]. Nevertheless, there still exist some structural differences between the sHSP 16.5 protein and α-crystallin. It is well known that small heat-shock proteins (sHSPs) form a structurally divergent protein family with members present in *Archaea*, *Bacteria*, and *Eukarya* [12,13]. All members of the sHSP family are characterized by the presence of a homologous sequence of about 80 residues, which has been denoted as the α-crystallin domain [3,14,15]. This domain is preceded by an NH_2_-terminal domain, which is highly variable in size and sequence, and is followed by a short, poorly conserved COOH-terminal extension. For all species, the secondary structure of the NH_2_-terminal domain is mainly an unordered structure while the COOH-terminal domain is rich in β-sheet structure. Based on NMR analysis, the COOH-terminal 8 and 10 residues of αA- and αB-crystallin, respectively, occur as solvent-exposed random coils [9]. Overall, previous studies suggested that a highly flexible COOH-terminal extension in mammalian sHSPs including αA- or αB-crystallin is required for full chaperone activity [16].

Since the αB subunit of α-crystallin is a more universal and authentic member of the sHSP family than αA-crystallin, we have previously focused on αΒ-crystallin and shown that COOH-terminal lysine truncation increases thermostability and enhances chaperone-like function of porcine αB-crystallin. In this study we have further truncated peptide segments from the COOH-terminal end of recombinant porcine αB-crystallin and constructed several site-specific mutants with the aim of providing insights into the structure-functional correlation on this important and novel molecular chaperone purported to play diverse roles in various pathological conditions including cataracts, cancers and neurodegenerative diseases [17,18].

## Methods

### Construction of expression vector

The wild-type αB-crystallin vector was constructed as described previously [19]. The mutant DNA fragments were made by polymerase chain reaction (PCR). The designed primers used for the construction of recombinant αB-crystallin and its various deletion plus COOH-terminal replacement mutants are listed in Appendix 1; Table S1. The forward primer contains an ATG initiator codon and an Nde I restriction site. The reverse primers were complementarily overlapped with the 3^’^-end of the coding region containing the Hind III site linked to the translational stop codon. The truncated nucleotide fragments were generated by PCR amplification of the corresponding segments in the open-reading frame of the porcine αB-crystallin expression vector. After twice-digesting with Nde I and Hind III, the product was purified and ligated into Nde I/Hind III sites of the isopropyl-β-D-thiogalactopyranoside (IPTG)–inducible *E. coli* expression vector pET21a^(+)^ (Novogen Inc., an Affiliate of Merck KGaA, Darmstadt, Germany).

### Expression and purification of porcine αB-crystallin and its mutants

*E. coli* strain BL21 (DE3) was transformed with expression constructs. The cells were incubated at 37 °C until the culture reached an optical density of 0.6 at OD_600nm_. Protein expression was incubated with additional IPTG to a final concentration of 1 mM. Four hours after incubation, cells were harvested and resuspended in 8 M urea and lysed by ultrasonication. After ultrasonication, the mixture was then centrifuged for 20 min at 20,000x g to remove any remaining insoluble debris. Soluble recombinant proteins were purified by TSK HW-55 gel filtration, followed by C4 reverse-phase HPLC. The purified proteins were lyophilized, and then analyzed by electrospray mass spectrometry to confirm their correct molecular masses of these subunits.

### Refolding and reconstitution of αB-crystallin and its mutants

Recombinant proteins were solubilized in 8 M urea individually, and then loaded onto gel filtration column (TSK HW-55). The fractions of proteins were then collected and concentrated. Concentrations were determined by an optical method based on extinction coefficients of amino-acid chromophores [20].

### Isoelectric focusing

The gel used in isoelectric focusing was non-denaturing 5% polyacrylamide, which contains no urea (Novex IEF Gel pH 3-10; Invitrogen, Carlsband, CA). After electrophoresis the gel was fixed in the buffer of 0.7 M trichloroacetic acid, 0.14 M 5-sulfosalicylic acid and stained with Coomassie blue. The approximate p*I* of proteins in the isoelectric focusing gel was estimated from p*I* calibration kits (p*I* 3-10; Amersham Pharmacia Biotech, Piscataway, NJ). The p*I* markers for the gel (top to bottom) were trypsinogen (p*I* 9.30), lentil lectin (p*I* ~8.15), myoglobin-basic band (p*I* 7.35), myoglobin-acid band (p*I* 6.85), human carbonic anhydrase B (p*I* 6.55), bovine carbonic anhydrase B (p*I* 5.85), α-lactoglobulin A (p*I* 5.20), soybean trypsin inhibitor (p*I* 4.55), and amyloglucosidase (p*I* 3.50).

### Circular dichroic spectra

CD spectra were performed on a JASCO J-715 spectropolarimeter (JASCO International Co., Tokyo, Japan). The structural change with respect to temperature was performed on J-715 spectropolarimeter connected to a NESLAB (Freehold, NJ) RTE-111 water bath thermal controller. The molar ellipticity at 217 nm was recorded every 0.5 °C, with a rate of temperature increase of 30 °C per hour, from 20 °C to 80 °C. Samples were 1.8x10^-5^ M in the buffer of 10 mM Na_2_HPO_4_, 2 mM KH_2_PO_4_, 3 mM KCl, and saturated with NaF, pH 7.4. The far-UV CD spectra were based on the mean of 5 accumulations with a 0.1 cm light-path cell. The near-UV CD spectra were based on the mean of 10 accumulations with a 1 cm light-path cell.

### Molecular mass analysis

Wild-type αB-crystallin and its mutants were dissolved in 50% acetonitrile containing 1% acetic acid to make a final concentration of 0.1 μM. The sample was then analyzed in an LCQ mass spectrometer (Finnigan, San Jose, CA) at an infusion rate of 5 μl/min. The spectra were analyzed with a software program (LCQ BioWorks, Victor, NY) supplied from the manufacturer.

### Gel-permeation FPLC

Multimeric sizes of wild-type αB-crystallin and its mutants were estimated on an analytical Superose-6 HR 10/30 prepacked column. High molecular mass standards (Amersham Pharmacia Biotech AB, Uppsala, Sweden) were used for calibration. The concentrations of wild-type αB-crystallin and its mutants were adjusted to the same concentration of 3.6x10^-5^ M and 1 ml sample each was applied to the column. The flow rate was 0.5 ml/min.

### Dynamic light scattering

Scattering experiments were performed on a DLS-700 dynamic light-scattering spectrophotometer (Otsuka Electronics Co., LTD., Tokyo. Japan). A He-Ne Laser operating at a wavelength of 632.8 nm was used as the light source. Protein samples in the buffer containing 50 mM Tris-HCl, 0.1 M NaCl, pH 8.0 were filtered through 0.45 μm filter membrane and incubated at 25 °C for 24 h. The concentrations of protein samples were adjusted to a concentration of 7.17x10^-5^ M determined by the aforementioned method.

### Assays of chaperone activity under non-thermal and thermal stresses

Insulin was used as a substrate for non-thermal chaperone activity assay. Bovine pancreas insulin was dissolved in 10 mM Na_2_HPO_4_, 2 mM KH_2_PO_4_, 3 mM KCl, and 0.1M NaCl, pH 11 and then rapidly adjusted pH of the solution to 7.4 with 10 mM Na_2_HPO_4_, 2 mM KH_2_PO_4_, 3 mM KCl, and 0.1M NaCl, pH 6.0. Fresh stock solution of insulin was made daily. Freshly prepared dithiothreitol (DTT) solution was used for the reduction of the insulin interchain disulfide with or without the addition of αB-crystallin or its mutant crystallins. The final concentration of DTT was 0.02 M. The turbidity of protein solution was recorded by its absorbance at 360 nm. Purified porcine βL- and γ-crystallins were used as substrates for thermal chaperone activity assays. The porcine βL- and γ-crystallins were purified as described in our previous reports [19,21,22]. Proteins were dissolved in a PBS buffer and the concentrations were determined before assays. The chaperone activity was assayed at given temperatures. Various molar ratios of substrate to αB-crystallin or its mutant crystallins were also used to compare their chaperone activities. Rabbit muscle aldolase (Sigma-Aldrich, St. Louis, MO) was used as the substrate for thermal aggregation assays at different temperatures. Freshly prepared aldolase solution (in PBS buffer) was used for the chaperone-activity assay.

### Thermal stability of recombinant αB-crystallin and its mutants

The assayed proteins were prepared in a PBS buffer containing 10 mM Na_2_HPO_4_, 2 mM KH_2_PO_4_, 3 mM KCl, and 0.1 M NaCl, pH 7.4. Each protein sample with the same concentration of 36 μM was heated in a temperature range of 20 to 80 °C, and the turbidity change was continuously monitored by absorbance at 360 nm.

## Results

### Expression, purification and characterization of recombinant αB-crystallin and mutants

**Figure 1:**
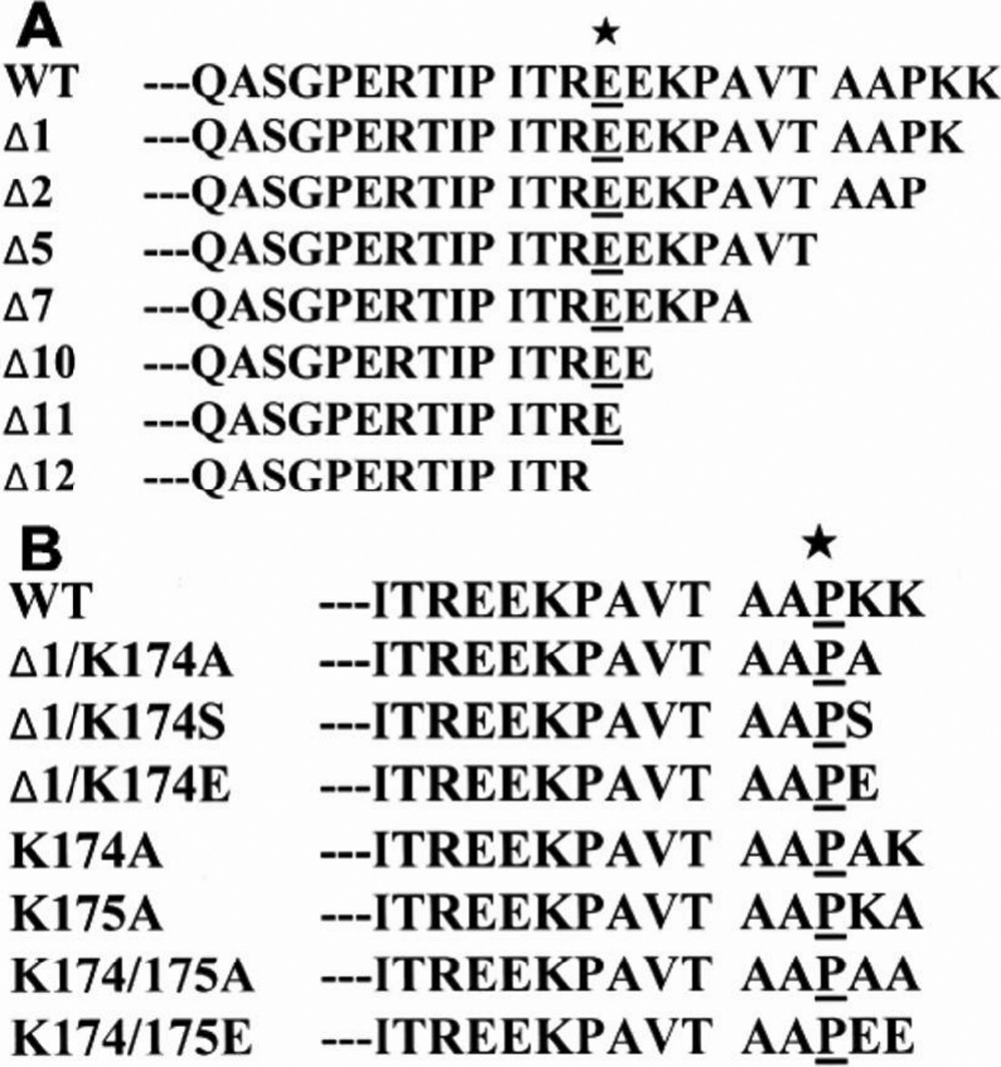
The COOH-terminal sequences of αB-crystallin and its mutants. **A**: The amino acid sequences from Q151 to the COOH-terminal of wild-type (WT) αB-crystallin and its truncated mutants. Various truncated mutants of porcine αB-crystallin have been constructed in order to study the functions of the COOH-terminal extension fragment of αB-crystallin. The amino acid (E164) of these mutants is underlined and marked with an asterisk. **B**: The amino acid sequences from I160 to the COOH-terminus of wild-type αB-crystallin and its mutants. Various mutants were constructed to study effects of amino-acid substitutions at position 174 and 175. The amino acid (P173) of these mutants is underlined and marked with an asterisk.

**Figure 2:**
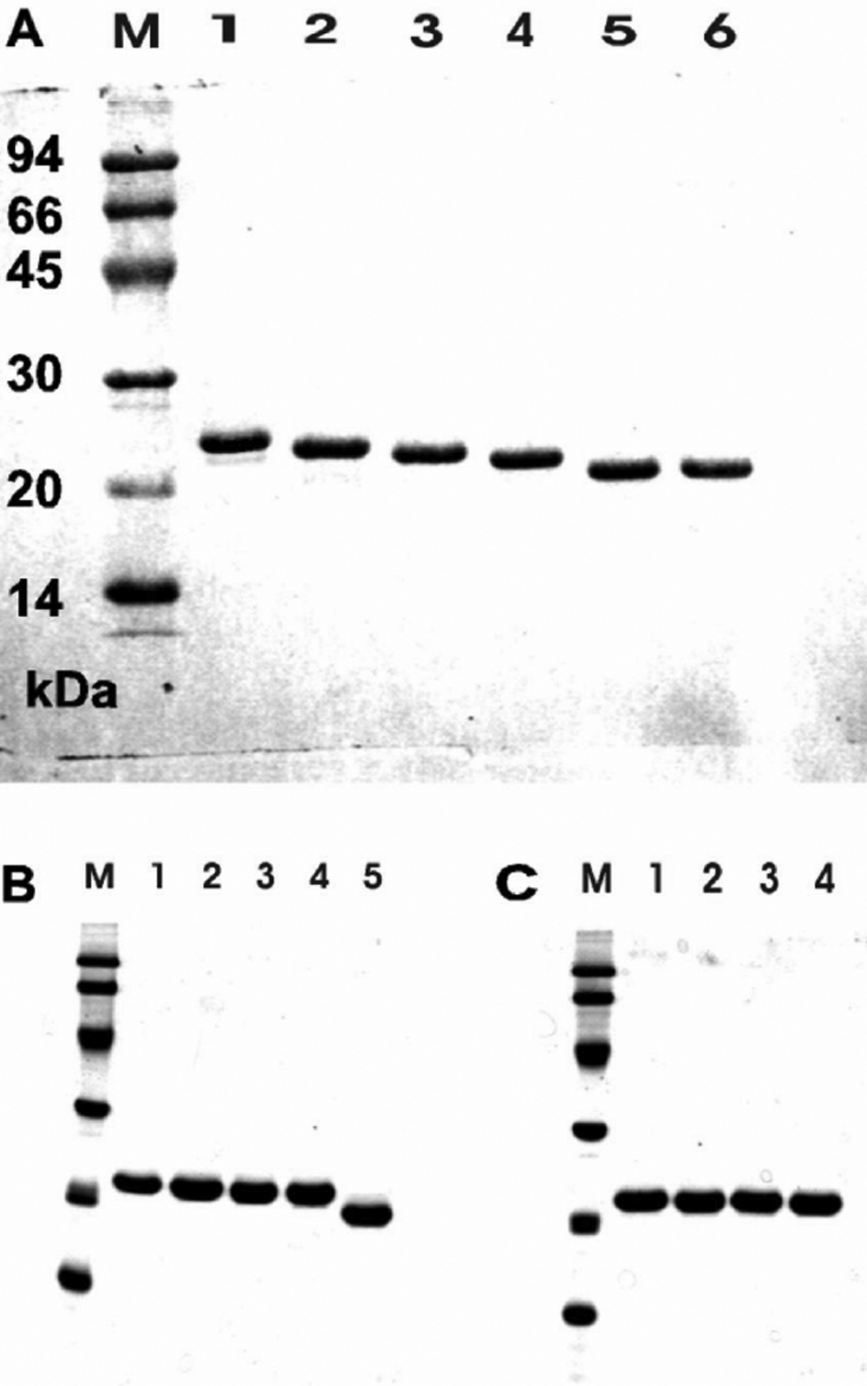
SDS-gel electrophoresis of the purified wild-type αB-crystallin and its mutants. αB-Crystallin and its mutant proteins were purified and the purity was checked by SDS-PAGE. Lane M, molecular mass markers. The masses of reference marker proteins are 94 kDa, 66 kDa, 45 kDa, 30 kDa, 20 kDa, and 14 kDa (from top to bottom of the gel), respectively. **A**: Lane 1, WT; Lane 2, Δ2; Lane 3, Δ5; Lane 4, Δ7; Lane 5, Δ10; and Lane 6, Δ12. **B**: Lane 1, Δ1; Lane 2, Δ1/K174A; Lane 3, Δ1/K174S; Lane 4, Δ1/K174E; and Lane 5, Δ11. **C**: Lane 1, K174A; Lane 2, K175A; Lane 3, K174/175A; and Lane 4, K174/175E. Coomassie brilliant blue-stained SDS gel shows essentially pure proteins for these crystallins (1-3 μg protein per lane).

**Figure 3:**
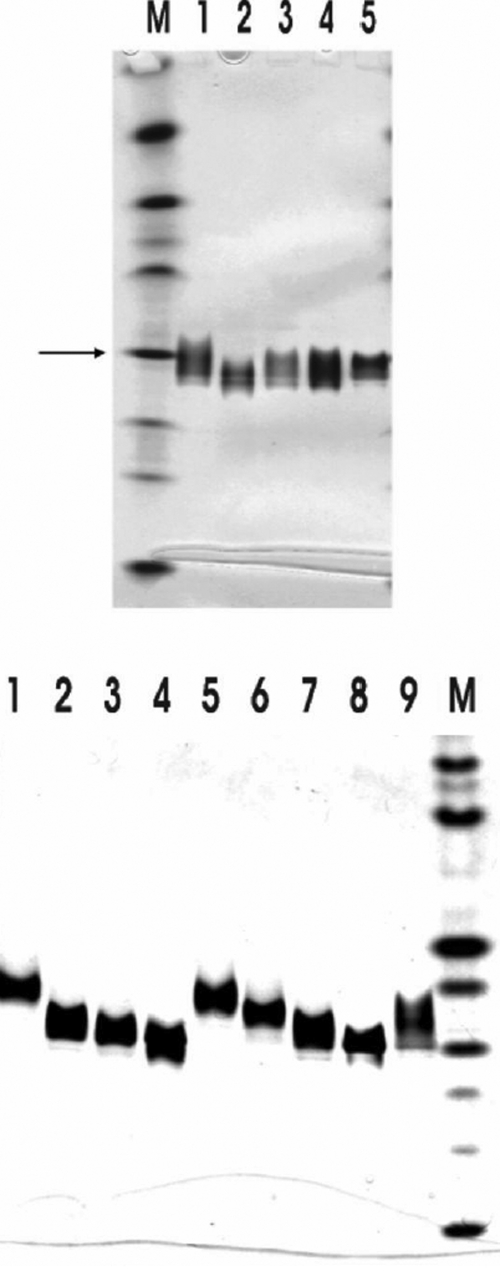
Isoelectric focusing of purified wild-type αB-crystallin and its mutants under native conditions. The approximate p*I* of isoelectric focusing gel was estimated from a p*I* calibration kit (p*I* range 3-10). The arrow indicated the position of bovine carbonic anhydrase B with a p*I* of about 5.85. **A**: Lane 1, WT; Lane 2, Δ2; Lane 3, Δ5; Lane 4, Δ7; and Lane 5, Δ10. **B**: Lane 1, Δ1; Lane 2, Δ1/K174A; Lane 3, Δ1/K174S; Lane 4, Δ1/K174E; Lane 5, K174A; Lane 6, K175A; Lane 7, K174/175A; Lane 8, K174/175E; and Lane 9, Δ11. Lanes M in **A** and **B** are p*I* calibration marker proteins. 3 μg of protein was used per lane and stained by Coomassie brilliant blue.

Various truncated mutants of porcine   αB-crystallin have been constructed to study the function of COOH-terminal extension segment of αB-crystallin (Figure 1A). These constructs were transformed in *E. coli* strain BL21 (DE3) and overexpressed in the presence of IPTG. Soluble recombinant proteins were purified by TSK HW-55 gel filtration, followed by reverse-phase HPLC (C4). Recombinant αB-crystallin and mutants were dissolved in 8 M urea, and refolded by eluting in a column of TSK HW-55(F). The recombinant αB-crystallin and mutants were thus reassociated and refolded after removal of urea. The purity of these proteins was checked by SDS-PAGE (Figure 2A). The deletion mutants Δ2, Δ5, and Δ7 show similar solubility with recombinant αB-crystallin. The Δ10 mutant showed a lower solubility than wild-type αB-crystallin when measured at low temperature; however, the Δ12 mutant showed even worse solubility and stability under ambient temperature. We also constructed various mutants by replacing amino acids to study the effects of COOH-terminal lysines (Figure 1B). The purity of these proteins was also checked and confirmed by SDS-PAGE (Figure 2B,C). The p*I* values of recombinant αB-crystallin and mutants were shown in Figure 3A,B. The approximate pH of the isoelectric-focusing gel was estimated from p*I* calibration kits. The p*I* value of the recombinant αB-crystallin corresponded to about that of bovine carbonic anhydrase B (p*I* 5.85). All mutants show p*I* values locating between 5.85 (bovine carbonic anhydrase B) and 6.55 (human carbonic anhydrase B). Recombinant αB-crystallin and mutants show distinct focusing zones with broad distribution of p*I* instead of sharp bands characteristic of p*I* marker proteins. The anomaly of broad distribution of p*I* values for recombinant αB-crystallin and its mutants may be due to their inherent polydisperse properties in their surface charges, and the existence of a rapid equilibrium of association-dissociation behavior associated with crystallin aggregation during electrophoresis.

### Secondary and tertiary structures of truncated mutants

Recombinant αB-crystallin and deletion mutants Δ2, Δ5, Δ7, and Δ10 all showed a similar β-sheet secondary structure to that reported previously as revealed in far-UV CD spectra (Appendix 1; Figure S1A) [22]. The near-UV CD spectra of recombinant αB-crystallin, and Δ2, Δ5, Δ7, and Δ10 were also similar (Appendix 1; Figure S1B). The intrinsic fluorescence spectra of recombinant αB-crystallin, Δ2, Δ5, Δ7, and Δ10 were all found to show emission maxima at 339-340 nm (Appendix 1; Figure S2), manifest of a largely hydrophobic microenvironment for the buried tryptophan residues [23]. However, the red-shift emission maxima Trp at 339-340 nm were observed indicating that Trp residues are relatively exposed in αB crystallin as compared with β- or γ-crystallin (Emax at 330 nm). We have also studied surface hydrophobicity differences among recombinant αB-crystallin and mutants by using the extrinsic fluorescent ANS probe as described before [22,24]. The ANS binding assays also showed similar surface hydrophobicity for recombinant αB-crystallin and mutants (Δ2, Δ5, Δ7, and Δ10; Appendix 1 Figure S3). Overall, recombinant αB-crystallin and all deletion mutants Δ2, Δ5, Δ7, and Δ10 possess similar secondary and tertiary structures.

### Quaternary structure and molecular sizes of truncated mutants

The aggregate sizes of recombinant αB-crystallin and mutants were estimated by analytical gel-permeation chromatography on Superose-6 HR FPLC column (Table 1). The molecular mass of wild-type αB-crystallin was estimated to be 550 kDa. The molecular masses of deletion mutants Δ1, Δ2, Δ5, Δ7, Δ10, and Δ11 were 400, 370, 500, 460, 450, and 480 kDa, respectively. Truncated mutants appear slightly smaller in molecular size than wild-type αB-crystallin. All truncated mutants show similar half-bandwidth elution peaks, indicative of a similar polydisperse property for these crystallins. Dynamic light scattering (DLS) measurements also revealed that the size of truncated mutants were slightly smaller than wild-type αB-crystallin (αB: 15.7±3 nm; Δ2: 13.1±3 nm; Δ5: 14.9±4 nm; Δ7: 15.1±4 nm; Δ10: 13.8±3 nm). Deletion mutant Δ12 is very susceptible to protein aggregation after protein expression and refolding from gel-permeation chromatography, leading to the difficulty of obtaining its size measurement.

**Table 1:** Molecular masses of subunits and native aggregates of wild-type αB-crystallin and its mutants.

**Protein**	**Subunit (Da)**	**Native aggregate (kDa)**
WT	20131	550
Δ1	20001	400
Δ2	19873	370
Δ5	19634	500
Δ7	19433	460
Δ10	19136	450
Δ11	19008	480
Δ12	18879	n.d.
Δ1/K174A	19942	420
Δ1/K174S	19957	330
Δ1/K174E	20001	370
K174A	20070	620
K175A	20071	570
K174/175A	20013	570
K174/175E	20131	630

The subunit masses were analyzed by using an LCQ mass spectrometer and native molecular masses determined by analytical gel-permeation FPLC. In the table, n.d. indicates not determined.

### Comparison of the chaperone activity of truncated mutants

**Figure 4:**
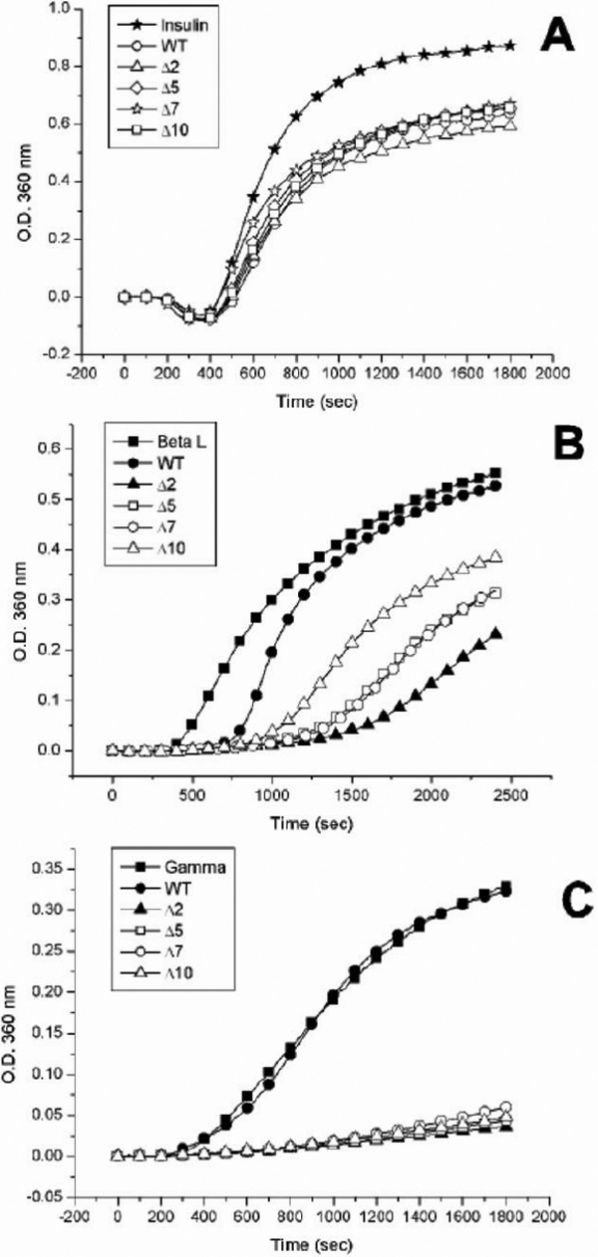
Comparison of chaperone activities of various αB-crystallin and deletion mutants against chemical and thermal denaturation. **A**: Inhibition of DTT-induced insulin B chain aggregation by wild-type αB-crystallin and its truncated mutants. The mutant proteins (Δ2, Δ5, Δ7, and Δ10) all show similar chaperone activities with wild-type αB-crystallin at 37 °C in a molar ratio (chaperone/insulin) of 1:16. The final concentration of bovine pancreas insulin is 59.3 μM. **B**: Inhibition of thermal denaturation of porcine βL-crystallin by wild-type αB-crystallin and its truncated mutants. βL-crystallin was used as a substrate for chaperone-activity assays at 60 °C. Mutant proteins (Δ2, Δ5, Δ7, and Δ10) all show higher chaperone activity than wild-type αB-crystallin in a molar ratio of (chaperone/βL-crystallin) of 2:7, with Δ2 showing highest activity among 4 deletion mutants. The final concentration of porcine βL-crystallin is 6.2 μM. **C**: Chaperone-activity assays using porcine γ-crystallin as a substrate. The wild-type αB-crystallin and its truncated mutants were heated at 65 °C in a molar ratio of 1:1 (chaperone/γ-crystallin). The mutant proteins (Δ2, Δ5, Δ7, and Δ10) all show better chaperone activities than wild-type αB-crystallin which exhibits no chaperone activity for heat denaturation of γ-crystallin. The final concentration of porcine γ-crystallin is 2.6 μM.

**Figure 5:**
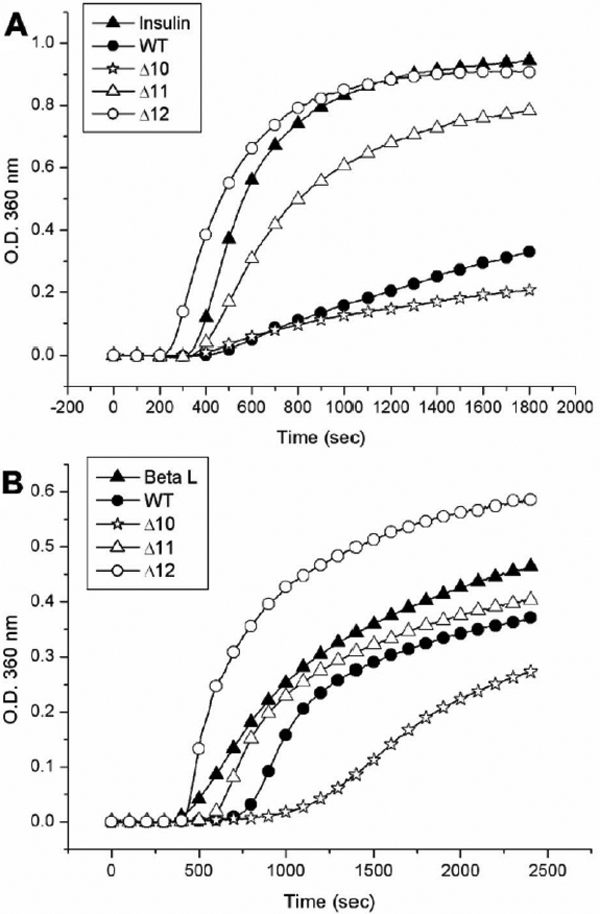
Comparison of chaperone activities of wild-type αB-crystallin and its truncated mutants under chemical and thermal denaturation. **A**: Inhibition of DTT-induced insulin B chain aggregation by wild-type αB-crystallin and its truncated mutants. The chaperone activities of Δ10 and wild-type αB-crystallin against chemical denaturation of insulin at 38 °C were similar in a molar ratio of 1:11 (chaperone/insulin). The mutant protein (Δ11) shows poor chaperone activity and Δ12 shows almost no protective activity under identical conditions. The final concentration of bovine pancreas insulin after mixing is 52.4 μM. **B**: Inhibition of thermal denaturation of porcine βL-crystallin by wild-type αB-crystallin and its truncated mutants. The mutant protein (Δ10) shows the best chaperone activity among four proteins tested at 59 °C in a molar ratio of 2:7 (chaperone/βL-crystallin). Wild-type αB-crystallin and Δ11 show poor chaperone activity under identical conditions, whereas Δ12 shows no protective activity. The final concentration of porcine βL-crystallin is 4.3 μM.

To distinguish whether the αB-crystallin and mutants have different chaperone activity under chemical denaturants, we assayed the chaperone-like activity of these mutants by using insulin as a substrate. At a molar ratio of 1:7.4 (chaperone/insulin), the truncated mutants (Δ2, Δ5, Δ7, and Δ10) showed similar chaperone activity to that of wild-type αB-crystallin at 37 °C. Under this condition, all of them showed almost complete inhibition of insulin B chain aggregation (Appendix 1; Figure S4). At a molar ratio of 1:16 (chaperone/insulin), truncated mutants (Δ2, Δ5, Δ7, and Δ10) also show similar chaperone activity to wild-type αB-crystallin at 37 °C (Figure 4A). On the other hand, by using alcohol dehydrogenase as a substrate to study the chaperone activity of these truncated mutants at 37 °C, the truncated mutants Δ2, Δ5, and Δ7 showed similar chaperone activity to wild-type αB-crystallin while Δ10 showed only a slight loss of chaperone activity. If alcohol dehydrogenase was substituted by porcine βL-crystallin as a substrate for heat denaturation similar to our previous reports [21,22], all truncated mutants also showed similar chaperone activity to wild-type αB-crystallin at a molar ratio of 1:1 (chaperone/βL-crystallin). However, when the molar ratio of βL-crystallin to these chaperones was increased, differences in chaperone activity of these mutants performed at 60 °C could be found. At a molar ratio of 1:3.5 (chaperone/βL-crystallin), Δ2 possessed the best chaperone activity among these mutants (Figure 4B). Mutants Δ5 and Δ7 were found to show similar chaperone activity with each other whereas Δ10 showed the least chaperone activity among these mutants. In this study we have also tried γ-crystallin as a substrate and assayed at 66 °C. At a molar ratio of 1:1 (Figure 4C), Δ2, Δ5, Δ7, and Δ10 show similar chaperone activity among one another. However, wild-type αB-crystallin was found to show little or no chaperone activity at such a high temperature. We also compared the chaperone activity of truncated mutants (Δ10, Δ11, and Δ12) with wild-type αB-crystallin. When insulin is used as a substrate at a molar ratio of 1:11 (chaperone/insulin) and assayed at 38 °C, these 3 truncated mutants show different chaperone activity (Figure 5A), with Δ10 showing similar or slightly better chaperone activity than wild-type αB-crystallin, Δ11 and Δ12 showing little or no chaperone activity. When we adopted βL-crystallin as the substrate and increased the assay temperature to 59 °C, the wild-type αB-crystallin, Δ11 and Δ12 were all found to lose most of their chaperone activity at a molar ratio of 1:3.4 (chaperone/ βL-crystallin) (Figure 5B). However, the truncated mutant Δ10 still maintained about 50% chaperone activity.

### Structural stability of recombinant αB-crystallin and truncated mutants

**Figure 6:**
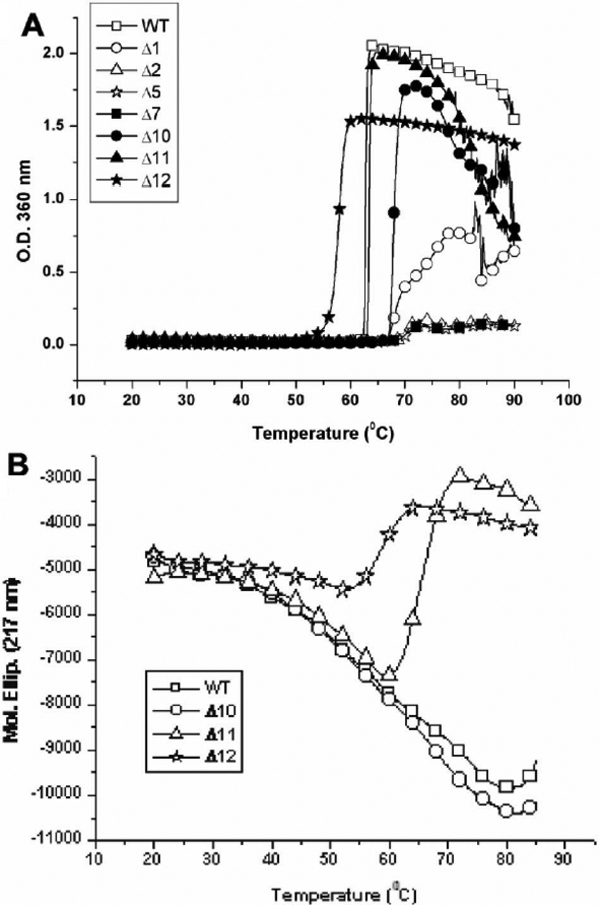
Thermal stabilities of wild-type αB-crystallin and its mutants. The turbidity change after heating was measured at 360 nm. Each sample (0.5 ml) of the same concentration (36 μM) was heated from 20 °C to 80 °C. Wild-type αB-crystallin and Δ11 are susceptible to aggregation and show serious light scattering (turbidity) at about 62 °C. Mutants Δ2, Δ5, and Δ7 show protein aggregation when heated to 70 °C. Mutant Δ1 becomes turbid near 67 °C and Δ10 is also susceptible to aggregation when heated to 67 °C, which displays greater light scattering than Δ2, Δ5, and Δ7. Mutant Δ12 is least stable as compared to other mutants and susceptible to aggregation at 57 °C. **B**: Conformational change of crystallin unfolding with heating. The molar ellipticity change at 217 nm of far-UV CD spectra, characteristic of protein β-sheet secondary structure was recorded with increasing temperature from 20 °C to 80 °C. Wild-type αB-crystallin and Δ10 show similar smooth change in molar ellipticity without abrupt change in protein secondary structure. The mutant protein (Δ11) shows a conformational transition around 65 °C, whereas Δ12 shows such a transition around 57 °C indicative of its susceptibility to unfolding at a lower temperature.

In Figure 6A, we studied the protein stability by incubating recombinant αB-crystallin and truncated mutants at the same concentration in a temperature range between 20 °C and 90 °C. Wild-type αB-crystallin solution became turbid at 62 °C, while Δ2, Δ5, and Δ7 solutions resisted higher temperatures and showed a slight turbidity at about 70 °C. Mutant Δ10 became turbid at 67 °C, whereas Δ12 turned turbid at 57 °C (Figure 6A). Mutants Δ1 and Δ11 were also found to be stable up to 67 °C and 62 °C, respectively. These thermostability tests suggest that the removal of COOH-terminal residue (Lys) in Δ1 and the 11th residue (Glu) from the COOH-terminal end in Δ11 appears to play opposite roles in the structural stability of deletion mutants under heat stress.

Since the secondary structure of αB-crystallin consists of mainly β-sheet structure, we studied the change of molar ellipticity at 217 nm by circular dichroism (CD) upon heating from 20 °C to above 80 °C (Figure 6B). We found a similar temperature-denaturation curve of αB-crystallin to that of a previous report [25]. In Figure 6B, deletion mutants Δ1, Δ2, Δ5, Δ7 (Data not shown), and Δ10 showed almost identical temperature-denaturation curves with wild-type αB-crystallin, with a decrease in molar ellipticity characteristic of a loss of β-sheet secondary structure upon heating from 40 °C to 60 °C. We compared the temperature-denaturation curves between wild-type αB-crystallin, Δ11, and Δ12 deletion mutants. Mutant Δ11 showed a transition temperature of about 70 °C and Δ12 near 60 °C, which reflected the fact that the deletion of the 12-residue segment E164-K175 in Δ12 can cause a greater reduction of the structural stability of αB-crystallin than Δ11. This result was consistent with thermostability tests for Δ11 and Δ12 by heating experiments mentioned above (Figure 6A).

### Structural and activity comparison of different site-specific mutants at positions 174 and 175 of αB-crystallin

**Figure 7:**
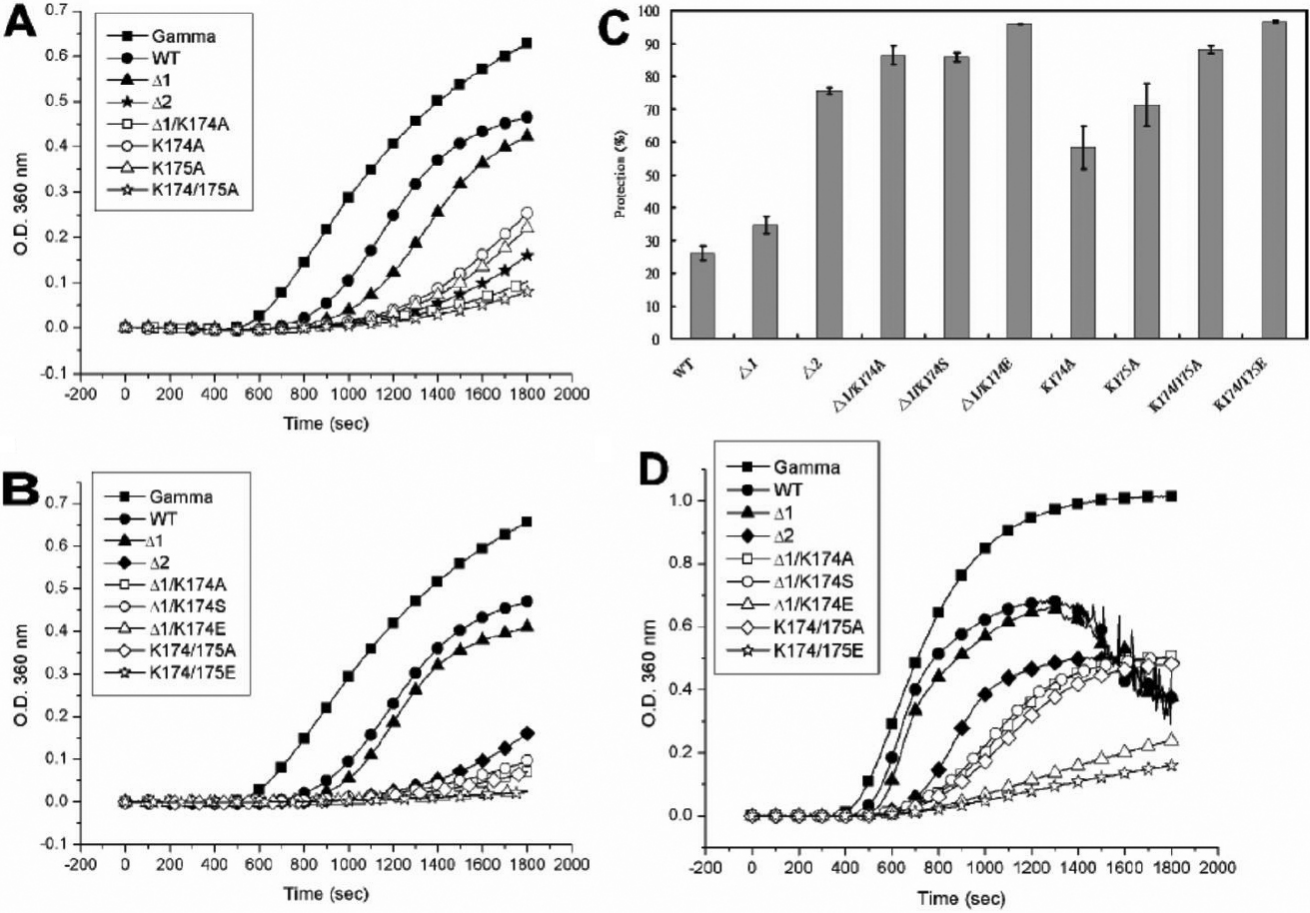
Comparison of chaperone activities of wild-type αB-crystallin and its mutants under thermal denaturation. **A**: Porcine α-crystallin was used as a substrate for chaperone-activity assays of wild-type αB-crystallin and mutants at 65 °C. Mutant proteins and wild-type αB-crystallin showed different chaperone activities at a molar ratio of 2:3 (chaperone/γ-crystallin). The scattering curves at 360 nm in the presence of chaperoning crystallins are shown as follows: control solution without chaperone (closed square), wild-type αB-crystallin (closed circle), Δ1 (closed triangle), Δ2 (closed asterisk), Δ1/K174A (open square), K174A (open circle), K175A (open triangle), and K174/175A (open asterisk). It is noted that K174/175A and Δ1/K174A show the highest activity among all mutants. **B**: Comparison of chaperone activities of wild-type αB-crystallin and mutants with different electrostatic amino acids under identical conditions as in **A**. The scattering curves at 360 nm in the presence of chaperoning crystallins are shown as follows: control solution without chaperone (closed square), wild-type αB-crystallin (closed circle), Δ1 (closed triangle), Δ2 (closed rhombus), Δ1/K174A (open square), Δ1/K174S (open circle), Δ1/K174E (open triangle), K174/175A (open rhombus), and K174/175E (open asterisk). **C**: Comparison of chaperone activity (percentage protection) of wild-type αB-crystallin and mutants. Wild-type αB-crystallin was shown to possess poor protective activity and K174/175E shown to possess the best protective activity among all proteins. The final concentration of porcine α-crystallin is 5.5 μM. **D**: Chaperone activities of wild-type αB-crystallin and its mutants under thermal denaturation at 70 °C. Porcine α-crystallin was used as a substrate for chaperone-activity assays of wild-type αB-crystallin and its mutants with different electrostatic amino acids at 70 °C in a molar ratio of 2:3 (chaperone/γ-crystallin). The scattering curves at 360 nm in the presence of chaperoning crystallins are shown as follows: control solution without chaperone (closed square), wild-type αB-crystallin (closed circle), Δ1 (closed triangle), Δ2 (closed rhombus), Δ1/K174A (open square), Δ1/K174S (open circle), Δ1/K174E (open triangle), K174/175A (open rhombus), and K174/175E (open asterisk). Both Δ1/K174E and K174/175E show the best protective activity among all mutants under these conditions. The final concentration of porcine α-crystallin is 5.5 μM.

In order to investigate the roles of lysine174 and lysine175, we constructed various mutants and analyzed their chaperone activities at high temperature. At a molar ratio of 1:4 (chaperone/βL-crystallin) at 60 °C, the double mutant, K174/175A, showed an almost complete inhibition of βL-crystallin aggregation (Appendix 1; Figure S5). Mutant Δ1 showed slightly better chaperone activity than wild-type αB-crystallin. Both single-replacement mutants, K174A and K175A, showed better chaperone activities than Δ1 and wild-type αB-crystallin. Mutants Δ1/K174A and K174/175A showed better chaperone activities than Δ2. The results indicate that two COOH-terminal lysines do not appear to play positively enhancing roles regarding chaperone activity of αB-crystallin. We then compared the chaperone activities of various mutants by changing electrostatic-charge states at the COOH-terminal end (Appendix 1; Figure S5). Mutants Δ1/K174A, Δ1/K174S, Δ1/K174E, K174/175A, and K174/175E all show better chaperone activities than Δ2, indicating again that the positive lysine residues may not contribute much to the chaperone activity. When we increased the temperature to 65 °C and used γ-crystallin as a substrate at a molar ratio of 2:3 (chaperone/γ-crystallin), similar results to βL-crystallin were observed (Figure 7). These results are summarized in Figure 7C as percentages of protection, (I_γ_-I_B_)/I_γ_x100, where I_γ_  is the intensity of scattered light for α-crystallin without chaperone protein in chaperone assays, and I_B_ is the intensity of scattered light in the presence of αB-crystallin or various mutants. Wild-type αB-crystallin showed about 26% protection whereas Δ1/K174E and K174/175E showed about 96% protection under the same assay conditions. Mutant Δ1 showed only 34% protection and Δ2 about 75% protection. Most prominently Δ1/K174A, Δ1/K174S, and K174/175A each showed about 87% protection. It is of interest to find that K175A showed 71% and K174A 58% protection, emphasizing that replacing lysine at position 174 contributes much less in increasing chaperone activity than at position 175. We further increase the temperature to 70 ^o^C and perform the same assay (Figure 7D). Wild-type αB-crystallin and Δ1 become extensively turbid after 20 min incubation in this assay. Mutants Δ1/K174A, Δ1/K174S, and K174/175A showed near 50% protection at 70 ^o^C. Both Δ1/K174E and K174/175E still showed good protective activities under this condition.

### Comparison of thermal stability and chaperone activity of αB-crystallin and its mutants at positions 174 and 175.

We also compared the thermal stability of wild-type αB-crystallin and its mutants at 70 °C for 15 min. All samples were in PBS with an identical concentration of 18 μM. Triplicate samples were incubated at 70 °C and their turbidities were measured at 340 nm. Both Δ1 and wild-type αB-crystallin became completely turbid after incubation at 70 °C for 15 min and other mutants showed better thermal resistance than wild-type αB-crystallin (Appendix 1; Figure S6).

**Figure 8:**
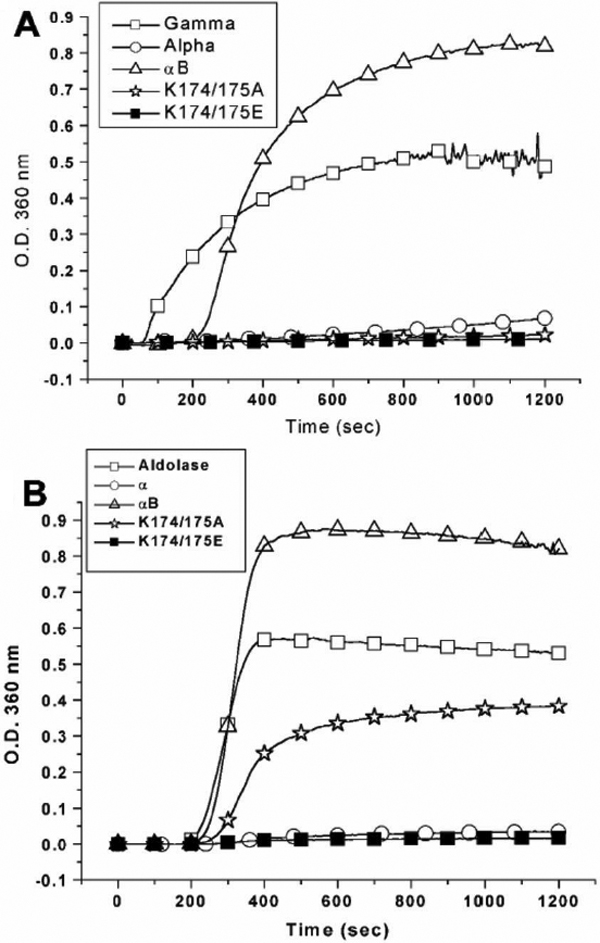
Comparison of chaperone activities of α-, αB-crystallin, K174/175A, and K174/175E. **A**: Comparison of chaperone activities under heat stress at 65 °C using porcine γ-crystallin as a substrate and in a molar ratio (chaperone/γ-crystallin) of 1:2.9. The final concentration of porcine γ-crystallin is 8.3 μM. **B**: Comparison of chaperone activities under heat stress at 70 °C using rabbit muscle aldolase as a substrate and in a molar ratio (chaperone/aldolase) of 1:1.7. The final concentration of porcine aldolase is 2.1 μM. The light-scattering (turbidity) curves at 360 nm in the presence of chaperoning crystallins are shown as follows: control solution without chaperone (open square), α-crystallin (open circle), αB-crystallin (open triangle), K174/175A (open asterisk), and K174/175E (closed square). It is noted that the chaperone activity of K174/175A can only be distinguished from that of α-crystallin and K174/175E using aldolase instead of γ-crystallin as a substrate in the chaperone assays under heat stress.

We have also compared the chaperone activities of α-, αB-crystallin, K174/175A, and K174/175E using porcine γ-crystallin as a substrate at a molar ratio of 1:2.9 (chaperones/γ-crystallin) under heating at 67 °C (Figure 8A). α-Crystallin, K174/175A, and K174/175E showed almost complete protection of γ-crystallin from thermal aggregation, while αB-crystallin failed to protect γ-crystallin under identical conditions (Figure 8A). Aldolase was also used as a substrate since it had been shown to be a better substrate for chaperone activity assay at high temperature. At a molar ratio of 1:1.7 (chaperone/aldolase), we compared chaperone activities of these four chaperones at 63 °C and 70 °C (Figure 8B). When we performed the assays at 63 °C (Appendix 1; Figure S7), αB-crystallin failed to protect aldolase from thermal aggregation. Surprisingly, K174/175A possessed 88% protection at 63 °C whereas K174/175E and α-crystallin showed 99% protection. Upon increasing the assay temperature to 70 °C, αB-crystallin also showed no chaperone activity and K174/175A decreased its activity to 28% protection while the other two maintained 99% protection (Figure 8B).

## Discussion

Like other sHSPs, both αA- and αB-crystallins provide thermotolerance when overexpressed in cells [26,27]. Outside the lens, αA-crystallin is only found at very low levels in some cell types, notably in the retina, kidney, and thymus [28-31]. In contrast, αB-crystallin is implicated in various important cellular processes, such as the suppression of protein aggregation [32], cytoskeletal protein dynamics [33,34], and cell protection [35]. αB-Crystallin is also overexpressed in some tissues of distinct pathological states, including retinoblastoma [36] and neurodegenerative diseases [37,38].

The major post-translational modification in human α-crystallin occurs in its COOH-terminal extension [39-42]. It was previously found that COOH-terminal truncated αA-crystallin by trypsin digestion showed a decreased ability to protect proteins from heat-induced aggregation using an in vitro assay [43]. The presence of an exposed COOH-terminal extension is in accordance with prevalent observations that the last 20 or so residues of the α-crystallin are especially liable to truncations and modifications by cellular proteases or other modification enzymes [44-48]. A previous report [49] also indicated that the COOH-terminal lysines of αB-crystallin are required for protection against ischemic damage in cardiomyocytes. The deletion of these two lysines produced a significant decrease in the molecular size of the native aggregate (Table 1), and this alteration was reported to diminish its stabilization of the microtubular cytoskeleton. The chaperone activity, determined with ADH and βL-crystallin as target proteins, was increased in human αA1-172 and decreased in αA1-168 and αA1-162 [50].

Previous literature abounds with reports characterizing sHSPs and various αA- or αB-crystallin mutants, often with inconsistent and contradictory findings. Since we have previously cloned and expressed porcine αB-crystallin [19], it is very useful to apply site-specific mutagenesis for deriving the structure-function correlation of this molecular chaperone. In this study, we have focused on the comparison of the structural thermostability and chaperone activity of αB-crystallin and its recombinant site-specific mutants, plus various COOH-terminal truncated mutants. In contrast to the report of Takemoto et al. [43], it is very intriguing to find that some of COOH-terminal truncated mutants (Δ2, Δ5, Δ7, and Δ10 in Figure 6) with higher thermostability actually perform better as molecular chaperones than the intact αB-crystallin. Plater et al. [51] also reported that mutations to COOH-terminal lysines of αB-crystallin reduced the chaperone-like activity of this crystallin. However, these authors adopted native α-crystallin that is composed of αA and αB with vastly different chaperone activity as a reference and control protein for activity comparison, which could lead to ambiguous conclusions regarding the structure-functional roles of these two lysine residues. In order to clear some discrepancy and contradictory results among different groups, we have directly used wild-type αB-crystallin as a control for a more meaningful activity comparison among various truncated or site-directed mutants.

To investigate the effect the length of COOH-terminal extension has on αB-crystallin chaperone function, we compared the chaperone activities of truncated mutants (Δ10, Δ11, and Δ12) with wild-type αB-crystallin. Chaperone activity assays based on insulin B chain aggregation induced by DTT showed that the truncated mutant Δ10 possesses similar chaperone activity to wild-type αB-crystallin (Figure 5A). Under similar assay conditions, the truncated mutant Δ11 showed less chaperone activity than wild-type αB-crystallin, whereas Δ12 showed no chaperone activity at all. Chaperone assays based on thermal denaturation of βL-crystallin showed wild-type αB-crystallin and Δ11 possess little chaperone activities (Figure 5B). On the other hand, the truncated mutant Δ10 still preserves about 50% chaperone activity whereas Δ12 shows no chaperone activity under similar heating conditions. In summary, the maximal truncated length in COOH-terminal extension of αB-crystallin to preserve chaperone function should be about ten amino acids. The result is in accord with that obtained from NMR analysis [9], which indicated that the COOH-terminal 8 and 10 residues of αA- and αB-crystallin, respectively, occur as solvent-exposed random coils.

Recombinant αB-crystallin, Δ2, Δ5, Δ7, Δ10, and Δ12 showed similar near-UV CD spectra, which indicated that these proteins have similar tertiary structure. The intrinsic fluorescence spectra of recombinant αB-crystallin, Δ2, Δ5, Δ7, and Δ10 were also similar; all of them showing emission maxima at 339-340 nm (Appendix 1; Figure S2). The emission maxima of Trp residues inside these proteins implied that Trp residues might be buried in the hydrophobic microenvironment. The ANS binding assays also showed similar surface hydrophobicity of these mutants (Δ2, Δ5, Δ7, and Δ10) with wild-type αB-crystallin (Appendix 1; Figure S3). The available data appeared to show COOH-terminal extension adopted no preferred conformation and extended outside the domain core of αB-crystallin. It has been proposed that the COOH-terminal extension segments in α-crystallin play an important role in solubilizing the protein and the formation of HMW complex during the process of chaperoning action [52]. It may bear some relevance to the observation that the deletion mutant Δ12 lost its solubility in water. In addition to acting as a solubilizer, the COOH-terminal extension of αB-crystallin may also play an important role in the stabilization of αB-crystallin assemblies. Our results of thermostability tests provided experimental evidence that the COOH-terminal extension indeed plays a critical role in the stabilization of αB-crystallin (Figure 6). Deletion of E164-K175 (Δ12) greatly reduces the structural stability of αB-crystallin. Recently, Treweek et al. [53] showed that K174/175A human αB-crystallin has better thermostability than wild-type human αB-crystallin, which is consistent with our study. Figure 7A shows that porcine αB-crystallin does not require two positive-charge lysines in protecting γ-crystallin from thermal aggregation. The positive-charge lysines may interfere with the chaperone activity at this site. Substitution of αB-crystallin Lys174–175 with glutamate leads to better chaperone activity than alanine- or serine-mutants (Figure 7C). It is noteworthy that Δ1/K174E and K174/175E showed remarkable chaperone activity at high temperature (70 °C, Figure 7D). Wild-type αB-crystallin and Δ1 become turbid after incubation at 70 ^o^C for 15 min. K174A and K175A show slightly higher turbidity than others (Appendix 1: Figure S6). It is to be noted that pH value of the PBS buffer used was 7.4 whereas p*I* values of these mutants lied between 5.85 and 6.55 (Figure 3), indicating that precipitation of proteins was caused by thermal denaturation instead of isoelectric precipitation of αB-crystallin. The results also indicated that COOH-terminal lysines may be the main residues to cause the aggregation of αB-crystallin at high temperature. In contrast, Δ1/K174E and K174/175E showed remarkable thermostability and chaperone activity at high temperature. The results suggest that charge repulsion of COOH-terminal lysines and some positive-charge groups of αB-crystallin may decrease the thermostability of αB-crystallin. On the other hand, charge attraction of COOH-terminal glutamates and these positive-charge groups of αB-crystallin can promote thermostability of K174/175E mutant.

Recently, the crystal structure of a eukaryotic wheat sHSP 16.9 was solved, revealing the mode of interaction between α-crystallin domain and flanking extensions leading to the assembly of sHSP into a dodecameric double disk similar to most prokaryotic chaperonin proteins [54]. The ability of the COOH-terminal extension to build different assemblies of oligomeric aggregates may stem from a hinge between  9 and 10 strands, thus allowing the angle between the α-crystallin domain and the COOH-terminal extension to vary by 30° and providing some mobile flexibility of COOH-terminal segments. Such a hinge mechanism may contribute to the size polydispersity observed in the assemblies of most sHSPs and α-crystallin subunits. The elimination of two positive Lys–Lys residues from the COOH-terminal segment may decrease charge-charge interaction of COOH-terminal coil with other parts of αB polypeptide chains, thereby increasing thermostability. However, the elimination of more than 10 residues from the COOH-terminal segment may decrease the ability of the terminal coil to hold neighboring subunits, thus decreasing thermostability and chaperone activity.

In great contrast to the report that mutations to the COOH-terminal lysines greatly reduced the chaperone-like activity of αB-crystallin [51], our results clearly show that mutations to COOH-terminal lysines actually enhance the chaperone-like activity of αB-crystallin. Finally, we have shown that native α-crystallin behaves differently from αB-crystallin. For example, αB-crystallin is unstable at temperatures higher than 62 °C, whereas α-crystallin is stable even at higher temperatures [21,55]. Herein we compared directly various mutants with wild-type αB-crystallin and showed that all site-specific mutants at lysine 174 and 175 possess better chaperone activities than wild-type αB-crystallin. Comparison of chaperone activities between α-, αB-, K174/175A, and K174/175E crystallins (Figure 8A) revealed that α-crystallin, K174/175A, and K174/175E can protect γ-crystallin from thermal aggregation while αB-crystallin fails to protect γ-crystallin under identical conditions. We also used aldolase as a substrate for chaperone activity assay (Figure 8B), showing that K174/175A mutant possesses better chaperone activity than αB-crystallin even though it still shows lower chaperone activity than α-crystallin. However, K174/175E shows similar chaperone activity with that of α-crystallin. In conclusion, our detailed characterization clearly demonstrated that both length and electrostatic charge of the COOH-terminal segment play crucial roles in governing the structural stability and chaperone activity of αB-crystallin.

## Appendix 1.

Supporting Information for COOH-terminal truncations and site-directed mutations enhance thermostability and chaperone-like activity of porcine αB-crystallin. To access the data, click or select the words “Appendix 1.” This will initiate the download of a compressed (pdf) archive that contains the file.
